# Applications of UV–Visible, Fluorescence and Mid-Infrared Spectroscopic Methods Combined with Chemometrics for the Authentication of Apple Vinegar

**DOI:** 10.3390/foods12061139

**Published:** 2023-03-08

**Authors:** Cagri Cavdaroglu, Banu Ozen

**Affiliations:** Department of Food Engineering, Izmir Institute of Technology, 35430 İzmir, Türkiye; cagricavdaroglu@iyte.edu.tr

**Keywords:** vinegar, adulteration, UV–visible spectroscopy, infrared spectroscopy, fluorescence spectroscopy, chemometrics, spirit vinegar, acetic acid, partial least square discriminant analysis

## Abstract

Spectroscopic techniques as untargeted methods have great potential in food authentication studies, and the evaluation of spectroscopic data with chemometric methods can provide accurate predictions of adulteration even for hard-to-identify cases such as the mixing of vinegar with adulterants having a very similar chemical nature. In this study, we aimed to compare the performances of three spectroscopic methods (fluorescence, UV–visible, mid-infrared) in the detection of acetic-acid/apple-vinegar and spirit-vinegar/apple-vinegar mixtures (1–50%). Data obtained with the three spectroscopic techniques were used in the generation of classification models with partial least square discriminant analysis (PLS-DA) and orthogonal partial least square discriminant analysis (OPLS-DA) to differentiate authentic and mixed samples. An improved classification approach was used in choosing the best models through a number of calibration and validation sets. Only the mid-infrared data provided robust and accurate classification models with a high classification rate (up to 96%), sensitivity (1) and specificity (up to 0.96) for the differentiation of the adulterated samples from authentic apple vinegars. Therefore, it was concluded that mid-infrared spectroscopy is a useful tool for the rapid authentication of apple vinegars and it is essential to test classification models with different datasets to obtain a robust model.

## 1. Introduction

Vinegar is a product which can be produced from various raw materials, mostly belonging to plant origins, with sugar as the substrate using a double fermentation process (ethanol fermentation and acetification). Vinegar can be classified with respect to its raw materials or production systems. Common types, considering the raw materials used, include wine, fruit, spirit/white (produced from diluted ethanol), cereal, malt, honey and whey vinegars, and they are most commonly produced through either surface culture (traditional) or submerged culture methods. Compositions of vinegars vary with respect to the raw materials from which they are produced. The major constituent is acetic acid; however, they also have various organic acids including citric, formic, lactic, malic and succinic acids, alcohols, sugars (glucose and fructose), amino acids, volatile compounds and phenolic compounds. The presence of phenolic compounds such as gallic acid, catechin, vanillic acid, syringic acid, caffeic acid and volatiles including ethyl heptanoate, ethyl furoate, ethyl benzoate and sotolon have been determined in vinegars [1]. Regulations about vinegars generally involve the amounts of acetic acid and ethanol in the product, which can vary slightly from country to country. The Food and Drug Administration (FDA) of the USA specifies the level of acetic acid as 4 g/100 mL, while the levels of acetic acid and ethanol are 50 g/L and less than 0.5% in the Codex, respectively. The European Union set a minimum of 5% (*w*/*v*) acidity and a maximum of 0.5% (*v*/*v*) ethanol levels for vinegars. While some countries allow the mixing of vinegar with acetic acid, others do not [1].

A projected compound annual growth rate of approximately 1.6% is expected between 2021–2026 for the vinegar market [2]. The increase in the global demand for vinegar is a result not only of its increased use in the food industry but also of its expanding applications in the cleaning, healthcare and agricultural industries. Besides its antimicrobial and antioxidant properties, another factor causing the consumer interest in vinegars is studies that have uncovered the positive health effects of this product [3,4]. Different claims such as weight loss, laxative effects and blood glucose lowering effects for type-2 diabetes patients, some of which require further confirmation studies, have also been made, particularly for apple vinegar [5]. However, this increased interest has also resulted in a rise in different types of fraud practices surrounding this product. Food fraud is described as ‘any deliberate action of businesses or individuals to deceive others in regards to the integrity of food to gain undue advantage’, and it is stated that this definition includes ‘adulteration, substitution, dilution, tampering, simulation, counterfeiting, and misrepresentation’ in addition to others [6]. The rising demand of consumers for good-quality, safe and healthy foods goes in parallel with the increase in the sophisticated ways that fraudsters misrepresent/adulterate these food products. Chemically similar and cheaper replacements of products can be very challenging to detect; therefore, there is always a need for alternative methods to determine different types of food fraud.

Variations in raw materials, production methods and regulations regarding the definition of the product along with the levels of acetic acid and ethanol add up to difficulties in adulteration detection for vinegars. Various adulterants are mixed with authentic vinegars to obtain economic profit. Adulteration can be achieved by adding chemical acetic acid, spirit vinegar, coloring compounds such as caramel and by mixing different types of vinegars. The false labeling of regular vinegars as high-priced vinegars with a protected-designation-of-origin status (PDO) or mixing PDO vinegars with adulterants is also an authenticity problem. Besides the economic effects of mixing, the addition of acetic acid can have particularly negative consequences, since it contains more heavy metals [1].

The targeted and untargeted methods available for the detection of vinegar adulteration have been summarized in several reviews in the literature [7,8]. Targeted methods such as chromatographic measurements focus on specific compounds such as a particular organic acid, a pigment or a phenolic compound [9,10]. Although valuable information can be obtained from the analysis of products using this type of approach, it also has disadvantages, as the used methods require time-consuming steps of sample pre-treatments that mostly involve the use of chemicals. On the other hand, untargeted methods, depending on their working principle, provide data originating from the many compounds in the analyzed product. Spectroscopic techniques, used mostly as untargeted methods, have the advantages of being rapid and generating relatively less waste, and they produce fingerprints of the analyzed samples. They are also very suitable for use as sensors [11,12,13]. Since spectroscopic techniques produce a large number of variables, multivariate statistical analysis tools are commonly used to evaluate these data. These chemometric methods can be used in classifying samples or for the prediction of chemical properties. Various spectroscopic methods have been investigated for the authentication of vinegars in the literature. Near-infrared (NIR) spectroscopy has been used in the classification of vinegars with regard to their production methods, and vinegars produced with the submerged and Orleans methods have been successfully differentiated [14]. The separation of balsamic and traditional balsamic vinegars of Modena with respect to their ages was achieved through the evaluation of nuclear magnetic resonance (NMR) spectroscopic data with chemometric methods, partial least square discriminant analysis (PLS-DA) and naive Bayes approaches [15]. Various studies about vinegar authentication have also been focused on the discrimination of vinegars according to their origin, and spectroscopic methods including NIR, mid-infrared (mid-IR), fluorescence, UV–visible and NMR spectroscopies have been applied for this purpose [16,17,18,19,20,21]. UV–visible and fluorescence spectral data were evaluated with principal component analysis (PCA) and parallel factor analysis (PARAFAC) in the discrimination of vinegars with respect to the country of production [21]. Spanish PDO vinegars, “Vinagre de Jerez” and “Vinagre Condado de Huelva”, were characterized with mid-IR spectroscopy, and the data were analyzed with PCA [16]. The performances of several spectroscopic methods, namely mid-IR spectroscopy, NIR spectroscopy, excitation–emission multidimensional fluorescence spectroscopy and 1H nuclear magnetic resonance (1H-NMR) spectroscopy, were compared in the classification of Spanish PDO vinegars, namely Vinagre de Jerez, Vinagre de Condado de Huelva and Vinagre de Montilla-Moriles, and the data were treated with data fusion techniques [20].

Spectroscopic methods were also used in differentiating mixtures of vinegars and, as an example, detection of the adulteration of sherry vinegars with molasses, rice, cider and wine vinegars was investigated with laser diode fluorescence spectroscopy in conjunction with chaotic algorithms [22]. Excitation–emission fluorescence spectroscopy, on the other hand, was used in differentiating authentic Shanxi aged vinegars from this vinegar mixed with acetic acid in combination with chemometric methods, and a 100% discrimination was achieved [23]. Although there have been many studies focusing on the different aspects of vinegar authentication, the number of studies on the detection of spirit vinegar and synthetic acetic acid is limited. In an earlier study, mid-IR and UV–visible spectroscopies were used to detect the adulteration of grape vinegars with spirit vinegar and acetic acid, and both techniques in combination with PLS-DA and orthogonal PLS-DA (OPLS-DA) were found to be successful in identifying adulterated grape vinegars [24]. The current study compared three spectroscopic techniques (UV–visible, fluorescence and mid-infrared) for their potential in the authentication of apple vinegars considering two adulterants. There are a limited number of studies regarding the mixing of vinegars with spirit vinegar and acetic acid, and the detection of these adulterants poses a challenge due to their similar chemical nature to vinegar. More studies are required to investigate the effect of the type of vinegar on the performances of various spectroscopic techniques in combination with chemometric methods so that suitable analytical and chemometric methods can be chosen for adulteration detection.

This study was designed to test and compare the potentials of various spectroscopic methods, namely UV–visible, fluorescence and mid-infrared, in conjunction with chemometric methods for detecting mixtures of apple vinegars with spirit vinegar and synthetic acetic acid.

## 2. Materials and Methods

### 2.1. Vinegar Samples and Adulteration

Seventeen authentic apple vinegars were supplied by eleven trusted producers. Two batches were obtained from each of two producers and five batches were obtained from one producer while the other producers supplied one batch. Two adulterated sample sets were prepared: apple-vinegar/spirit-vinegar and apple-vinegar/acetic-acid mixtures. Each set had adulterant levels of 1, 5, 10, 20, 30, 40 and 50% (*v*/*v*). Glacial acetic acid used as an adulterant was diluted to a typical vinegar acetic acid level of 4% (*v*/*v*) before mixing with the vinegars. Eight apple vinegars were randomly chosen among seventeen vinegars to mix with two spirit vinegars and acetic acid separately, and one hundred and eighty-five adulterated samples were prepared.

### 2.2. Measurement of Quality Parameters

pH and Brix values of the authentic vinegars were determined with a pH meter (WTW, Weilheim, Germany) and a digital refractometer (Isolab, Wertheim, Germany), respectively. Total acidity expressed as a volumetric percentage was measured via titration analysis using sodium hydroxide [25]. A microscale Folin–Ciocalteu spectrophotometric assay was used in the measurement of the total phenolic content in terms of mg gallic acid/L of the authentic vinegars [26]. The total phenolic contents of the authentic apple vinegars were determined using a 5-point gallic acid standard curve.

### 2.3. Fluorescence Spectroscopy

Spectra of authentic and adulterated samples were collected with a fluorescence spectrophotometer (Thermo Scientific Varioskan, Fisher Scientific, Vantaa, Finland) at 320–800 nm with 1 nm intervals. Excitation wavelengths were 320, 330, 340 and 350 nm [27]. The best results were obtained at 320 nm. The slit width was 5 nm. Samples were diluted 5 times, and the spectra of 200 μL samples in a black 96-well flat bottom polystyrene plate (Isolab, Wertheim, Germany) were collected. Two spectra from each sample were averaged.

### 2.4. UV–Visible Spectroscopy

A total of 200 μL from all the samples diluted 5× with distilled water in 96-well flat bottom polystyrene plates (Isolab, Wertheim, Germany) was scanned in 200–550 nm range with a UV–vis spectrophotometer (Thermo Scientific Multiskan GO Microplate Spectrophotometer, Fisher Scientific, Vantaa, Finland). The average of two spectra for each sample was used in the statistical analyses.

### 2.5. Fourier Transform Infrared Spectroscopy

Mid-IR spectra of the samples were obtained with a Fourier transform infrared (FTIR) spectrophotometer with a horizontal ZnSe ATR accessory and a deuterated triglycine sulfate (DTGS) detector (Spectrum 100, Perkin Elmer, Waltham, MA, USA). The spectra were collected in 4000–800 cm^−1^ range with 128 scans and a 4 cm^−1^ resolution against an air spectrum. Two measurements were taken for each sample, and they were averaged.

### 2.6. Statistical Analysis

One of the unsupervised techniques, principal component analysis (PCA), was performed as a preliminary analysis. A discrimination trend between the authentic and adulterated samples in the scatter plot of the first and second principal components was observed; therefore, it was decided to continue with a higher-level multivariate analysis. Differentiation of the authentic and adulterated apple vinegars was conducted with two supervised chemometric methods, namely partial least square discriminant analysis (PLS-DA) and orthogonal partial least square discriminant analysis (OPLS-DA). PLS-DA and OPLS-DA are supervised multivariate classification techniques, and they convert data to a lower dimension through linear transformation. The authentic samples were defined as one class, and all the adulterated samples were assigned to another class. The raw and transformed data from the 3 spectroscopic techniques were used in the chemometric model building. Along with intensity values at different emission wavelengths for fluorescence spectroscopy, the absorption values of the samples at different wavenumbers and wavelengths for the mid-IR and UV–vis spectroscopy, respectively, were individually collected in column-wise vectors. After the collection of the data, individual observations were combined in a row-wise matrix prior to the multivariate analysis. The following data transformations were applied: first (FD), second (SD) and third (TD) derivatives, square, standard normal variate (SNV), multiplicative scatter correction (MSC) and Savitzky–Golay (SG). In addition, the following combinations of these transformations were also used: FD + SNV, FD + MSC, SD + SNV, SD + MSC, TD + MSC and TD + MSC. Every feature in the collected and transposed dataset was normalized using the scaling of 0–1, which is called a min-max normalization. Models were created using the ‘ropls package’ (Version 3.12) in the R programming language [28]. Two-thirds of the data were used for building the calibration models, while the external validation was conducted with the rest of the data. The samples were assigned to the calibration and validation sets using stratified random sampling [29]. The goodness of the classification models was evaluated using the number of latent variables (LV), R^2^ values for calibration (R^2^_cal_) and validation (R^2^_val_), root mean square of error (RMSE), sensitivity, specificity, correct classification rates for calibration and validation. Definitions of correct classification rates, sensitivity and specificity are provided in the literature [30]. Sensitivity was measured as the ratio of the true number of correctly identified apple vinegars to all the samples identified as apple vinegar and was calculated using:

$$Sensitivity=\frac{TP}{TP+FN}$$

where *TP* and *FN* are samples identified as true positive and false negative, respectively.

On the other hand, dividing the number of correctly identified adulterated samples to all the samples identified as adulterated provided the specificity:

$$Specificity=\frac{TN}{TN+FP}$$

where *TN* and *FP* are samples identified as true negative and false positive, respectively.

The correct classification rate was calculated by dividing the number of correctly determined samples to all the samples, and it was determined for both the calibration and validation sets as follows:

$$Correct\;classification\;rate=\frac{TP+TN}{Total\;number\;of\;samples}\times 100$$

## 3. Results and Discussion

Various properties of the authentic apple vinegars are shown in Table 1. The authentic vinegar samples had pH and Brix ranges of 2.74–2.99 and 0.6–5.3, respectively. The total acidity of these samples varied between 4.08 and 5.49%. The vinegars had total phenolic contents of 163.15–547.40 mg gallic acid/L. These measurements were in agreement with the values given in the literature [31].

### 3.1. Spectroscopic Profiles

**Figure 1:**
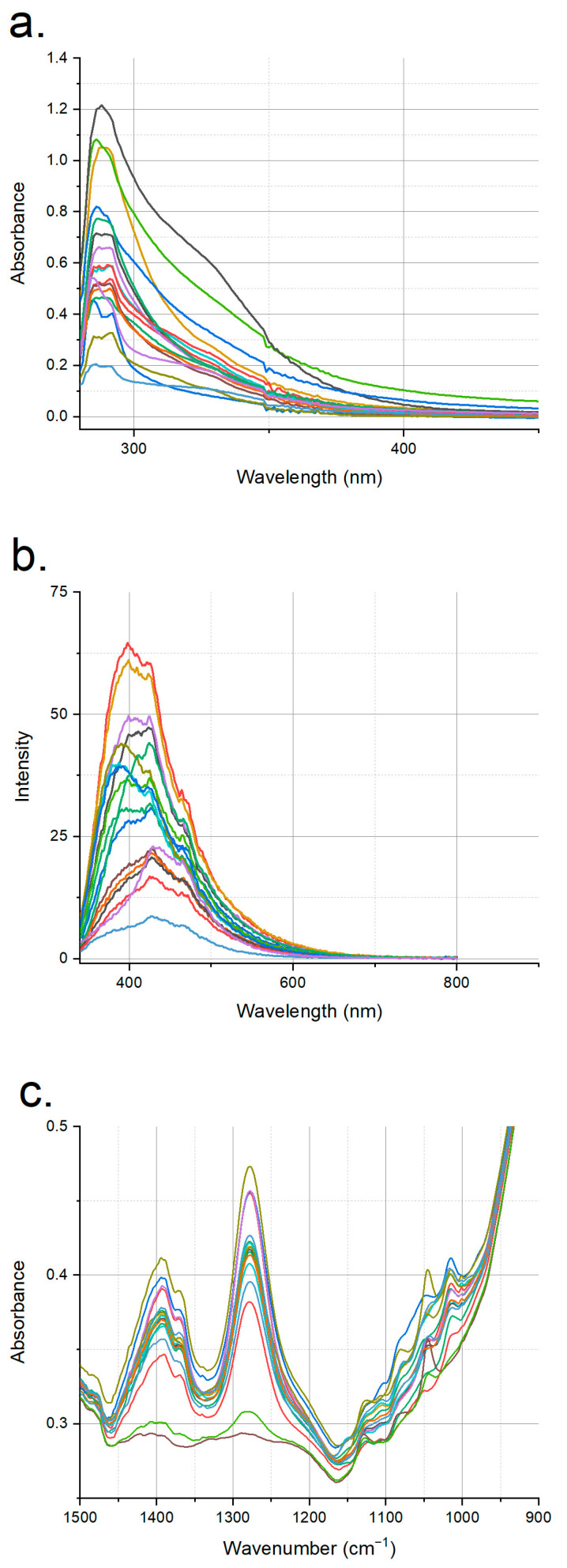
(**a**) UV–visible, (**b**) fluorescence and (**c**) mid-IR spectra of all authentic apple vinegars used in this study.

**Figure 2:**
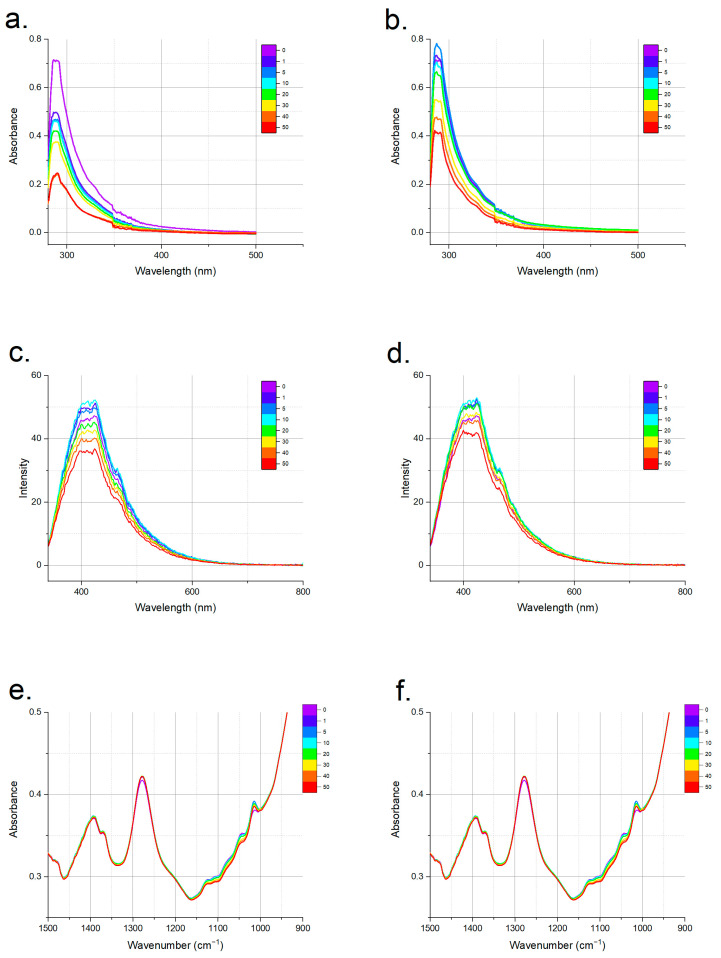
UV–visible spectra of (**a**) spirit-vinegar-added and (**b**) acetic-acid-added samples; fluorescence spectra of (**c**) spirit-vinegar-added and (**d**) acetic-acid-added samples; and mid-IR spectra of (**e**) spirit-vinegar-added and (**f**) acetic-acid-added samples vs. authentic apple vinegars for a sample set.

The authentic apple vinegars had strong double absorption peaks in 280–300 nm range of the UV–visible spectra (Figure 1a), and these peaks were associated with phenolic compounds, as reported in the literature [32,33]. The authentic vinegars had a wide absorption range in the UV–visible range, which was most probably due to their varying phenolic compositions, and this was confirmed by the measured total phenolic contents of the authentic apple vinegars, which were in the range of 163.15–547.4 mg gallic acid/L. The UV–visible, fluorescence and mid-IR spectra of an example set of adulterated spirit vinegar and acetic acid vs. authentic vinegars are shown in Figure 2. In general, the absorbance of the adulterated samples in 280–400 nm range decreased with increasing adulteration ratio due to the dilution of phenolic compounds with an adulterant (Figure 2a,b). This decrease was more obvious in the spirit-vinegar-adulterated samples, while the dilution effect was visible at around 20% for the acetic-acid-mixed samples for this particular sample set. Spirit vinegar is produced from bio-resources through fermentation, while acetic acid is a synthetic product without any ingredients from biological sources. Therefore, these differences in the adulterated sample spectra can be related with the sources of the adulterants.

For the authentic apple vinegars, a wide variation in intensity was also observed in their fluorescence spectra (Figure 1b). The spectra could be characterized by strong intensity peaks in 300–600 nm region. Phenolic compounds are designated as having fluorescent properties, and this range corresponds to the intensity due to these compounds [23,34]. The peak at around 470–500 nm was attributed to brown pigments, which can be produced by acetic acid bacteria [27]. As was observed in the UV–visible spectra, the fluorescence spectra of the authentic vinegar vs. the spirit vinegar adulterated apple vinegar and the authentic vinegar vs. acetic acid adulterated apple vinegar sample sets indicated a dilution effect but at higher concentrations compared to the UV–visible spectra (Figure 2c,d).

The mid-IR spectra were collected in 4000–800 cm^−1^ region; however, it is generally hard to see major differences if the full spectra are shown. Therefore, part of the spectra corresponding to 1500–800 cm^−1^ region are presented for all the authentic apple vinegars in Figure 1c. The mid-IR spectra of the adulterated and authentic samples had significant differences, especially in 1500–1000 cm^−1^ region (Figure 2e,f). The peaks in the 1400–1350 cm^−1^ region were attributed to –OH stretching of alcohol and organic acids [24], and the adulterated samples had a higher absorption in this region, as was expected. However, the absorption intensity decreased with respect to the ratio for the adulterated samples in 1150–1000 cm^−1^ region where absorption took place due to compounds such as sugars and phenolic compounds, and this decrease in the absorption intensity was also attributed to the addition of adulterants. Differences in the spectra obtained by these three spectroscopic methods were also evaluated by chemometric methods, which can reveal even small changes in the spectra that are not very visible, and this is especially useful for spectroscopic methods with large number of variables, as is the case in mid-IR spectroscopy.

### 3.2. Chemometric Analyses

A set of randomly chosen vinegars were adulterated with spirit vinegar and diluted acetic acid (4%) separately. The adulterated set contained both apple-vinegar/spirit-vinegar and apple-vinegar/acetic-acid mixtures, and PLS-DA and OPLS-DA chemometric models were constructed to differentiate the authentic apple vinegars from the mixtures. Separate models were not created for each adulterant since the nature of the adulterant would not be known in a more realistic scenario. Therefore, two classes were created as the authentic and adulterated sets. The whole collected spectral ranges of all the spectroscopic methods were used in the chemometric analyses.

**Figure 3:**
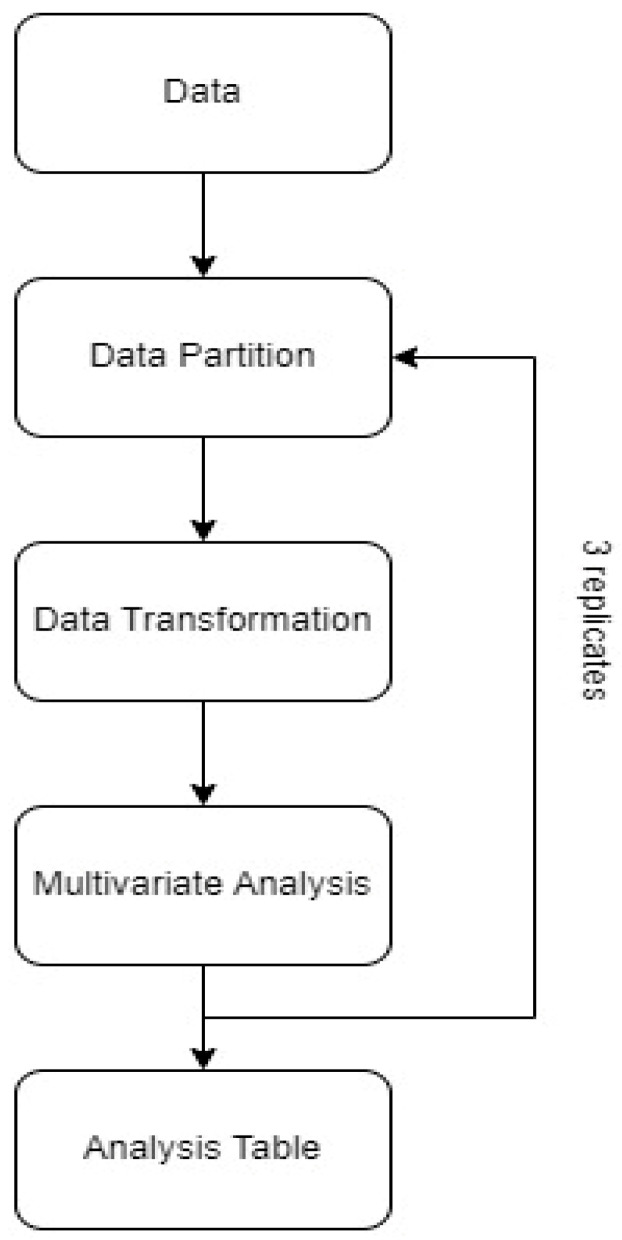
Flow chart of data analysis.

An improved approach was used in deciding on the best classification models (Figure 3). Both raw and transformed data, as indicated in Section 2.6, were used in generating the PLS-DA and OPLS-DA models. This procedure was repeated three times, and each time a new randomly chosen data set for calibration and validation was used. For each trial, the statistical performance parameters (LV, R^2^_cal_, R^2^_val_, RMSE, sensitivity, specificity, correct classification rates for calibration and validation) of the models were determined. The models which provided good and robust results for all the trials were designated as our final models. The purpose of this approach was to eliminate the effect of the samples in the model building. Therefore, the chosen robust models had high R^2^ values for the calibration and validation models, high correct classification rates for classification and validation sets, high sensitivity and specificity values and a low RMSE value regardless of the sample.

Furthermore, the models improved significantly when the samples with a 1% adulteration level were eliminated from the sample set. Since this is a very low level of mixing for the economic gain of fraudsters, the 1% samples were taken out from the sample set, and the models were constructed with the samples with higher adulteration levels. After the removal of the 1%-adulterated samples, the models were built with 107 samples and validated with 52 samples.

The only good model, which was built with the fluorescence spectra and had correct classification rates of 90% for calibration, 92% for validation, a sensitivity of 1 and a specificity of 0.92, belonged to the PLS-DA analysis of the MSC-transformed data (Table 2). As can be seen from Table 2, the sensitivity and specificity values were unacceptable for the second and the third sample sets. It was concluded that this transformation and any other transformations of the fluorescence spectra did not result in any good classification model for the differentiation of the authentic and adulterated vinegars when different calibration and validation sets were used in the second and the third runs. The same type of results was also obtained with the UV–visible spectral data. Although there were models with high correct classification rates for the validation models for the first sample set, similar results were not obtained in the second and the third runs with different sample sets. For example, the OPLS-DA model after SNV transformation of the data with a correct classification rate of 93% for calibration, a correct classification rate of 92% for validation, a sensitivity of 0.67 and a specificity of 0.94 was the best model (Table 2). However, this model with different sample sets did not result in any good specificity value, although these models had high correct classification rates. Therefore, it was concluded that the robustness of classification models had to be decided not only with the correct classification rates but also with the sensitivity and specificity values and that the models had to be checked with different sample sets.

**Figure 4:**
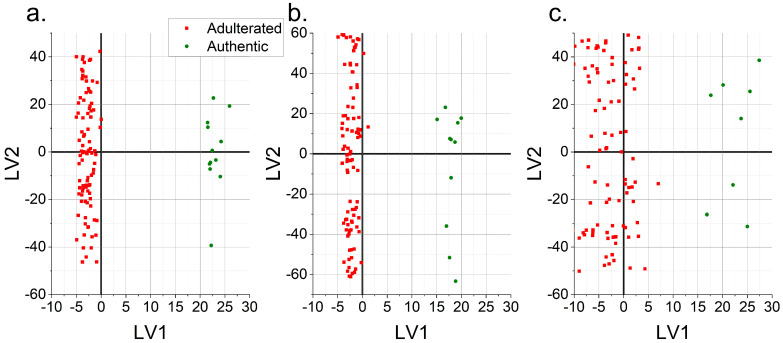
OPLS-DA score plots (LV1 vs. LV2) of Savitzky–Golay-transformed mid-IR data constructed with three different sample sets (**a**–**c**).

The same type of approach was also used for evaluating the mid-IR data. Six chemometric models constructed with the FTIR data resulted in robust models: the PLS-DA and OPLS-DA models of the raw data, the square-transformed data and the SG-transformed data. Table 3 shows the statistical measures related with the performance of these models for three different sets of samples. For each sample set, these models had a high sensitivity and specificity as well as high correct classification rates for calibration and validation. These models had very close performance parameters when they were created with different sample sets. For example, the OPLS-DA model generated with raw data had the same sensitivity value of 1 and specificity values of 0.92, 0.94 and 0.94 for each sample set. Both the PLS-DA and OPLS-DA models produced very similar results in terms of the performances of the models. As an example, the PLS-DA and OPLS-DA models of the SG-treated mid-IR data had the same sensitivity (1), specificity (0.92) and correct classification rate for validation (92%) with the first and the second sample sets (sensitivity: 1, specificity: 0.96, correct classification rate: 96%) (Table 3). All six models shown in Table 3 were quite satisfactory and could be used successfully in detecting the adulteration of apple vinegar with acetic acid and spirit vinegar. Score plots of the OPLS-DA model of the SG-transformed mid-IR data for three different sample sets are given in Figure 4. As can be seen from this figure, the authentic and adulterated samples could be accurately differentiated from each other with respect to the first LV regardless of the sample set. In addition, this study indicates the importance of constructing classification models with different sample sets so that a more robust and accurate model can be obtained. In addition, not only the correct classification rates but also the sensitivity and specificity values have to be considered in evaluating models.

Most studies about vinegar authentication using spectroscopic techniques have focused on differentiation with respect to the source or type of vinegar [15,16,17,18,19,20,21]. However, studies which investigated the determination of the mixing of different types of adulterants with vinegar also exist in the literature. An electronic nose system was used in detecting the addition of acetic acid and spirit vinegar to apple vinegar in conjunction with the use of PCA and an artificial neural network (ANN), and correct classification rates of 93.3% for acetic acid and 94.7% for synthetic vinegar were determined for the ANN models [35]. Laser diode fluorescence spectroscopy data were evaluated with various intelligent chaotic algorithms to detect the presence of molasses, rice, cider and white wine vinegars in sherry vinegar; as a result, relative errors in predicting the adulterant concentration as low as 1.4% were obtained [22]. One study which investigated the determination of glacial acetic acid in Shanxi aged vinegars used excitation–emission matrix fluorescence spectroscopy data in combination with various chemometric approaches, and a model with a correct classification rate of 84.2%, a sensitivity of 0.83 and a specificity of 0.85 was obtained [20]. Since the adulterated product was a special type and was aged, the larger compositional differences between the authentic and adulterated samples could be the reason for the better success rate in that study compared to our case. In another study, in which the detection of spirit-vinegar- and acetic-acid-adulterated grape vinegars were studied using UV–visible and FTIR spectroscopy, the models created with UV–visible data had a correct classification rate of 95.2%, a sensitivity of 0.857 and a specificity of 0.964, and the FTIR data resulted in a model with 96.7% correct classification rate, a sensitivity of 0.857 and a specificity of 0.982 [24]. The same success was not obtained for apple vinegar adulteration with spirit and acetic acid using UV–visible spectroscopy in the current study. This could be related to the pigment composition of the apple vinegars. The authentic apple vinegars used in this study had a wide phenolic content range, and this was also reflected in the UV–visible spectra of the authentic vinegars (Figure 1a) and hence in the models generated using these spectra. In addition, the type of phenolic compounds present in apple and grape vinegars are different. Since UV–visible spectroscopy measurements are based on absorption due to colored compounds, the types and amounts of these compounds could be associated with the difference in the success of UV–visible spectroscopic data in the detection of adulteration of apple and grape vinegars. However, the evaluation of the FTIR spectral data resulted in a very good differentiation of apple vinegar adulteration, and the results of this study are comparable with the results of a previous study done using grape vinegar [24]. While fluorescence and UV–visible spectroscopic measurements are based on the detection of fluorescent and colored compounds, respectively, FTIR spectral data can provide more compositional information of all the organic constituents of analyzed samples, and this could be the reason for the more satisfactory performance of this technique, regardless of the vinegar type.

## 4. Conclusions

The mixing of spirit vinegar and acetic acid with apple vinegar was investigated with UV–visible, fluorescence and mid-IR spectroscopy in combination with chemometric tools. Classification models used to separate the authentic and adulterated samples were created with testing models with three different data sets, and it was concluded that this step is important in choosing robust and accurate classification models. The performance of only the mid-IR spectroscopy was considered as successful in determining the presence of spirit vinegar and acetic acid in the apple vinegar, and models were able to determine adulteration with at least a correct classification rate of 92%, a sensitivity of 1 and a specificity of 0.92. Therefore, mid-IR spectroscopy in combination with a chemometric classification system can be used as a rapid analysis technique in determining the adulteration of apple vinegars.

## Figures and Tables

**Table 1:** Various properties of authentic apple vinegar samples.

Number of Samples	pH Range	Brix Range	Total Phenolic Content Range (mg Gallic Acid/L)
17	2.74–2.99	0.6–5.3	163.15–547.40

**Table 2:** Statistical measures of models generated using UV–visible and fluorescence spectroscopic data with three different data sets.

Statistical Measures *									
		LV	R^2^_cal_	R^2^_val_	RMSE	Sensitivity	Specificity	CC_cal_%	CC_val_%
MSC-transformed fluorescence data with PLS-DA									
	First sample set	3	0.96	0.11	0.303	1	0.92	90	92
	Second sample set	7	0.97	0.87	0.119	0.2	0.91	100	85
	Third sample set	4	0.96	0.43	0.243	NaN	0.9	94	90
SNV-transformed UV–visible data with OPLS-DA	First sample set	1 + 7	0.98	0.47	0.241	0.67	0.94	93	92
	Second sample set	1 + 7	0.99	0.46	0.243	0	0.9	93	88
	Third sample set	1 + 7	0.99	0.44	0.247	NaN	0.9	93	90

* LV: number of latent variables; RMSE: root mean square of error; CC_cal_%: correct classification rate for calibration; CC_val_%: correct classification rate for validation, NaN: cannot be calculated.

**Table 3:** Statistical measures of models generated using mid-IR data with three different data sets.

Statistical Measures *									
		LV	R^2^_cal_	R^2^_val_	RMSE	Sensitivity	Specificity	CC_cal_%	CC_val_%
PLS-DA									
Raw	First sample set	8	0.99	0.77	0.159	1	0.92	99	92
	Second sample set	10	0.99	0.85	0.128	1	0.94	100	94
	Third sample set	10	0.99	0.86	0.125	1	0.94	100	94
Square	First sample set	10	0.99	0.85	0.13	1	0.94	100	94
	Second sample set	10	0.99	0.84	0.135	1	0.94	99	94
	Third sample set	10	0.99	0.83	0.139	1	0.94	99	94
Savitzky–Golay	First sample set	8	0.99	0.75	0.165	1	0.92	99	92
	Second sample set	10	0.99	0.83	0.138	1	0.96	100	96
	Third sample set	10	0.99	0.84	0.134	1	0.94	100	94
OPLS-DA									
Raw	First sample set	1 + 8	0.99	0.84	0.133	1	0.92	99	92
	Second sample set	1 + 9	0.99	0.85	0.128	1	0.94	100	94
	Third sample set	1 + 9	0.99	0.86	0.125	1	0.96	100	96
Square	First sample set	1 + 8	0.99	0.81	0.143	1	0.94	100	94
	Second sample set	1 + 8	0.99	0.78	0.157	1	0.94	99	94
	Third sample set	1 + 7	0.00	0.83	0.139	1	0.96	99	96
Savitzky–Golay	First sample set	1 + 8	0.99	0.82	0.14	1	0.92	99	92
	Second sample set	1 + 9	0.99	0.83	0.138	1	0.96	100	96
	Third sample set	1 + 5	0.99	0.84	0.134	1	0.92	95	92

* LV: number of latent variables; RMSE: root mean square of error; CC_cal_%: correct classification rate for calibration; CC_val_%: correct classification rate for validation.

## Data Availability

Data will be available on request.
